# Eicosapentaenoic acid suppresses ocular inflammation in endotoxin-induced uveitis

**Published:** 2010-07-17

**Authors:** Misa Suzuki, Kousuke Noda, Shunsuke Kubota, Manabu Hirasawa, Yoko Ozawa, Kazuo Tsubota, Nobuhisa Mizuki, Susumu Ishida

**Affiliations:** 1 Department of Ophthalmology and Visual Science, Yokohama City University Graduate School of Medicine, Yokohama, Japan; 2 Laboratory of Retinal Cell Biology, Keio University Center for Integrated Medical Research, Tokyo, Japan; 3 Department of Ophthalmology, Keio University School of Medicine, Tokyo, Japan; 4 Department of Ophthalmology, Hokkaido University Graduate School of Medicine, Sapporo, Japan

## Abstract

**Purpose:**

To investigate the effect of eicosapentaenoic acid (EPA) on acute ocular inflammation in an animal model of endotoxin-induced uveitis (EIU).

**Methods:**

C57Bl/6 mice (6-week-old males) were orally treated with EPA at a dose of 50 mg/kg/day for 5 days. EIU was then induced in the animals by intraperitoneal injection of 160 µg lipopolysaccharide (LPS). Twenty-four hours after LPS injection, leukocyte adhesion to the retinal vasculature was evaluated by the concanavalin A lectin perfusion-labeling technique, and leukocyte infiltration into the vitreous cavity was quantified. Furthermore, the protein levels of monocyte chemotactic protein (MCP)-1, interleukin (IL)-6, intercellular adhesion molecule-1 and phospholyrated nuclear factor (NF)-κB p65 in the retina and retinal pigment epithelium (RPE)–choroid complex were examined by enzyme-linked immunosorbent assay (ELISA).

**Results:**

At 24 h after LPS injection, the EIU animals treated with oral EPA administration showed a significant decrease in leukocyte adhesion to the retinal vessels by 43.4% (p<0.01) and leukocyte infiltration into the vitreous cavity by 49.2% (p<0.05). In addition, EPA significantly reduced the protein levels of MCP-1 and IL-6 in the retina and the RPE-choroid complex. Furthermore, phosphorylation of NF-κB was suppressed by EPA treatment.

**Conclusions:**

Our data suggest that EPA inhibits multiple inflammatory molecules in vivo. EPA may become a novel strategy in the prevention and/or treatment of ocular inflammatory diseases.

## Introduction

Recent studies have elucidated that inflammation is one of the characteristic features of systemic diseases such as atherosclerosis, coronary heart disease, diabetes mellitus, and hypertension [1-4]. Plasma levels of C-reactive protein and pro-inflammatory cytokines, such as tumor necrosis factor (TNF)-α and interleukin (IL)-6, are elevated in subjects with essential hypertension, coronary heart disease and type 2 diabetes [5,6]. Furthermore, evidence is emerging that anti-inflammatory drugs ameliorate the conditions and/or delay the onset of these systemic diseases [7-9]. Consequently, it seems likely that prevention and/or suppression of systemic inflammation reduces the risks of these life-threatening diseases, and thus to that end much attention has been paid to a variety of types of candidate anti-inflammatory agents. One such promising type is that of the safe disease-modifying nutrients, which can be ingested over a long period without remarkable harm. For example, clinical studies have demonstrated that administering higher doses per bodyweight of fish oil beneficially modulated systemic inflammatory processes [10-12]. Moreover, epidemiological observations have revealed that the Inuit, who consume fish daily, have a lower incidence of autoimmune and/or inflammatory disorders compared with gender- and age-matched groups living in Denmark [13]. As a result of these investigations, fish oil has become recognized as an important dietary supplement for prevention of systemic diseases caused by underlying inflammatory responses.

Eicosapentaenoic acid (EPA) is one representative of the ω-3 polyunsaturated fatty acids (PUFA), which are highly contained in fish oil. EPA has been clinically used in patients with hyperlipidemia to lower serum lipid levels, and it has been shown to produce anti-inflammatory effects [14,15], which, taken together, suggest that the preventive or protective effects of fish oil in systemic diseases are, at least in part, attributed to EPA. It was shown, for instance, that EPA-rich fish oil ameliorates systemic human inflammatory diseases such as rheumatoid arthritis [16]. Similarly, EPA reduced the recurrence of aphtha in patients with Behçet disease, a cause also of uveitis [17]. In accordance with the clinical data, EPA decreased leukocyte chemotaxis, adhesion molecule expression, and production of pro-inflammatory cytokines in an animal model of systemic diseases [18,19]. Our group has also elucidated that EPA suppresses the formation of inflammation-induced neovascularization and choroidal neovascularization via suppression of pro-inflammatory cytokines [20]. Thus, accumulating data propose a protective benefit of EPA in ocular inflammatory diseases. However, despite the documented anti-inflammatory effects of EPA, the molecular mechanism(s) by which EPA modulates acute ocular inflammation is not well understood. In this study, we investigate EPA’s effects on ocular inflammation, using an established animal model, the endotoxin-induced uveitis (EIU) [21].

## Methods

### Endotoxin-induced uveitis and EPA treatment

Six-week-old C57Bl/6 mice (CLEA, Tokyo, Japan) were used. Animals were orally fed with either EPA (kindly given by the Mochida Pharmaceutical, Tokyo, Japan) at a dose of 50 mg/kg/day or vehicle solution (CMC: carboxymethylcellulose) using stomach sonde for 5 days, and then received a single intraperitoneal injection of 160 μg lipopolysaccharide (LPS) from *Escherichia coli* (Sigma-Aldrich, St. Louis, MO) in phosphate buffered saline (PBS). Control animals received intraperitoneal injections of the same volume of vehicle (300 µl of PBS). All animal experiments were approved by the Animal Care Committee of the Keio University School of Medicine and conducted in accordance with the ARVO Statement for the Use of Animals in Ophthalmic and Vision Research.

### Quantification of firm leukocyte adhesion

Mice were anesthetized with intramuscular injection of a mixture of 80 mg/kg Ketamine and 16 mg/kg Xylazine before surgical procedures. Leukocytes firmly adhering to the retinal vasculature were visualized and quantified by perfusion-labeling with fluorescein-isothiocyanate (FITC)-coupled concanavalin A lectin (Con A; Vector, Burlingame, CA), as described previously [22]. Briefly, the chest cavity was opened under deep anesthesia and a 27-gauge cannula was introduced into the left ventricle. Animals were perfused with 2 ml of PBS to remove intravascular content, including nonadherent leukocytes. Perfusion with Con A (2 ml) was then performed to label adherent leukocytes and vascular endothelial cells, followed by removal of residual unbound lectin with 2 ml of PBS perfusion. Afterwards, eyes were enucleated and retinas were flatmounted. Flatmounted retinas were imaged with an epifluorescence microscope (IX71; Olympus, Tokyo, Japan), and the total number of Con A-stained leukocytes per retina was counted in a masked fashion.

### Analysis of leukocyte infiltration into the vitreous cavity

Leukocyte infiltration into the vitreous cavity was analyzed, as described previously [22]. Briefly, 24 h after LPS injection, eyes were enucleated from the animals while under deep anesthesia and fixed in 4% PFA. Three 5 µm paraffin sections were prepared at a distance of 100 µm from each other with the middle section passing through the optic nerve. In the sections, the number of infiltrating cells in the vitreous cavity was counted and the averaged numbers were used for evaluation.

### Enzyme-linked immunosorbent assay (ELISA)

The retinal tissue and retinal pigment epithelium (RPE)-choroid complex of each mouse were carefully isolated and placed into 100 µl of lysis buffer (0.02M HEPES, 10% glycerol, 10 mM Na_4_P_2_O_7_, 100 μM Na_3_VO_4_, 1% Triton, 100 mM NaF, and 4 mM EDTA [pH 8.0]) supplemented with protease inhibitors. After the sonication, the lysate was centrifuged at 20,400× g for 15 min at 4 °C. The protein levels of monocyte chemotactic protein (MCP)-1, IL-6, and intercellular adhesion molecule (ICAM)-1 in the supernatant were determined with mouse MCP-1, IL-6, and ICAM-1 ELISA kits (R&D Systems, Minneapolis, MN).

The level of phosphorylated nuclear factor (NF)-κB p65 in the supernatant was also determined with the mouse pNF-κB p65 Sandwich ELISA kit (Cell Signaling, Danvers, MA), according to the manufacturer’s protocols. The tissue sample concentration was calculated from a standard curve and corrected for protein concentration.

### Statistics

All results are expressed as mean±SEM (standard error) with n-numbers as indicated. Student’s *t* test was used for statistical comparison between the groups. Differences were considered statistically significant at p<0.05.

## Results

### Impact of EPA on the leukocyte recruitment cascade during ocular inflammation

**Figure 1:**
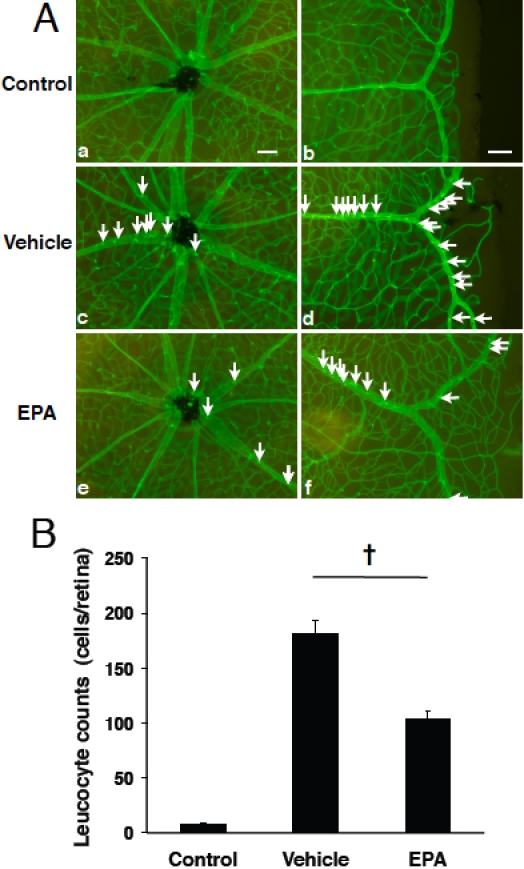
Impact of EPA on firm leukocyte adhesion in the retinal vessels during EIU. **A**: Representative micrographs of flatmounted retinas from normal control mice (a,b), vehicle-treated EIU mice (c,d) and EPA-treated (50 mg/kg BW) EIU mice (e,f) at 24 h after treatment and EIU-induction. Firmly adhering leukocytes in the retinal vasculature were visualized by perfusion with ConA. Arrows indicate firmly adhering leukocytes in the inflamed retinal vasculature (c-e). Scale bars=100 µm. **B**: Quantification of firm adhering leukocytes in the retinal vessels. Values are means±SEM (n=9 to 16). †p<0.01.

To study the effects of EPA on acute ocular inflammation, first we counted the number of leukocytes adhering to the vessels in the retinal flatmounts of EIU animals with or without EPA treatment. Control animals without LPS stimulation showed no or very few adhering leukocytes in the retinal vessels (8±4 cells/retina, n=9; Figure 1A,B). However, at 24 h after LPS injection, a large number of leukocytes firmly adhered to the retinal vessels in vehicle-treated animals (182±42 cells/retina, n=14; Figure 1A,B) compared to controls. By contrast, the EIU animals treated previously with oral EPA administration over a 5-day period showed a significant decrease in leukocyte adhesion to the retinal vessels by 43.4% (103±31 cells, n=16, p<0.01, Figure 1A,B) compared with the vehicle-treated EIU animals.

**Figure 2:**
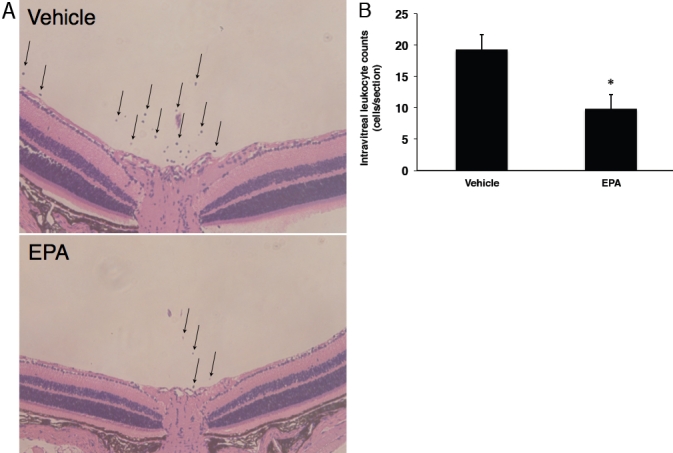
Impact of EPA on inflammatory leukocyte infiltration into the vitreous cavity. **A**: Representative micrographs of leukocyte infiltration into the vitreous of EIU mice with or without EPA administration. Arrows indicate the infiltrated leukocytes in the vitreous. **B**: Quantification of infiltrated leukocytes in the vitreous cavity of EIU animals treated with or without EPA administration. Values are means±SEM (n=8). *p<0.05.

Next, to study the impact of EPA on leukocyte infiltration into the vitreous cavity, we quantified the number of leukocytes in the vitreous of control versus EIU animals treated with either EPA or vehicle solution. Representative sections from EIU animals showed extravasated leukocytes around the optic disc (Figure 2A), while very few leukocytes were found in the vitreous cavity of normal animals (data not shown), as described previously [22]. Specifically and moreover, upon counting we confirmed a higher number of leukocytes to be in the vitreous of these EIU animals with vehicle-only treatment (19±2 cells/section, n=8; Figure 2B). However, compared to this group of animals, the number of infiltrating cells into the vitreous cavity of the EIU animals having undergone EPA treatment was significantly reduced by 49.2% (10±2 cells/section, n=8, p<0.05; Figure 2B).

### Impact of EPA on expression of inflammation-associated molecules in the retina

**Figure 3:**
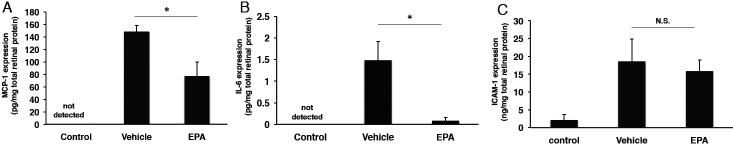
Role of EPA on expression of inflammatory mediators and endothelial adhesion molecules in the retina during EIU. Protein levels of MCP-1 (**A**), IL-6 (**B**), and ICAM-1 (**C**) in the retina from vehicle-treated normal control mice, vehicle-treated EIU mice, and EPA-treated EIU mice were measured by ELISA at 24 h after the treatment. Values are means±SEM (n=5 to 7). *p<0.05.

To further determine whether supplemental EPA may ameliorate ocular inflammatory responses during EIU, we also measured representative inflammation-associated molecules, i.e., MCP-1, IL-6, and ICAM-1, in the retina. The level of MCP-1 was undetectable in the retinal tissue of control animals (n=7; Figure 3A). In the EPA-treated animals, retinal MCP-1 (76.6±23.6 pg/mg total protein, n=7) was significantly decreased by 48.2% at 24 h after LPS injection, compared to the vehicle-treated EIU animals (148±10.2 pg/mg, n=6, p<0.05; Figure 3A). Similar to MCP-1, the level of IL-6 protein was also undetectable in the retinal tissue of control animals (n=6; Figure 3B). Whereas EIU animals showed the upregulation of IL-6 in the retina (1.5±0.4 pg/mg, n=6), EPA treatment significantly reduced the average level of retinal IL-6 (0.08±0.07 pg/mg, n=6, p<0.05; Figure 3B) by 94.5% at 24 h after LPS injection.

In contrast, protein levels of ICAM-1 were not significantly statistically different between vehicle-treated EIU animals (18.6±2.6 ng/mg, n=6) and EPA-treated EIU animals (15.8±1.4 ng/mg, n=5, p=0.378; Figure 3C).

### Impact of EPA on expression of inflammation-associated molecules in the RPE-choroid complex

**Figure 4:**
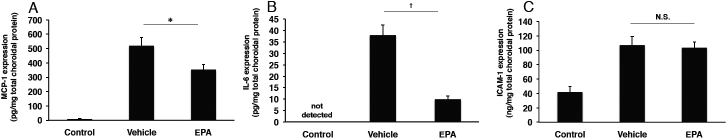
Role of EPA on expression of inflammatory mediators and endothelial adhesion molecules in the RPE-choroid complex during EIU. Protein levels of MCP-1 (**A**), IL-6 (**B**), and ICAM-1 (**C**) in the RPE-choroid complex from vehicle-treated normal control mice, vehicle-treated EIU mice and EPA-treated EIU mice were measured by ELISA at 24 h after the treatment. Values are means±SEM (n=4 to 8). ^†^p<0.01. *p<0.05.

To investigate whether supplemental EPA may suppress inflammatory molecules in the uveal tissue during EIU, we measured MCP-1, IL-6, and ICAM-1 in the RPE-choroid complex. Twenty-four hours after LPS-injection, the average level of MCP-1 in the RPE-choroid complex of the EIU animals was upregulated (519.0±57.3 pg/mg, n=8, p<0.01) compared with that of the controls (8.6±2 pg/mg, n=4; Figure 4A). In contrast, protein levels of MCP-1 in the RPE-choroid complex were significantly decreased by 31.9% in the EPA-treated animals (353.3±36.2 pg/mg, n=8, p<0.05; Figure 4A). As for IL-6 in the RPE-choroid complex, EPA-treated EIU animals showed a decrease in the average level (9.9±1.4 pg/mg, n=8) by 73.9% in comparison with EIU animals (38.0±4.5 pg/mg, n=8, p<0.01; Figure 4B). Similar to the retinal tissue, the level of IL-6 was undetectable in the RPE-choroid complex of control animals (Figure 4B).

The protein levels of ICAM-1 were not significantly statistically different between vehicle-treated EIU animals (106.9±4.2 ng/mg, n=8) and EPA-treated EIU animals (103.3±2.9 ng/mg, n=8, p=0.477; Figure 4C).

### Impact of EPA on suppression of NF-κB activation

**Figure 5:**
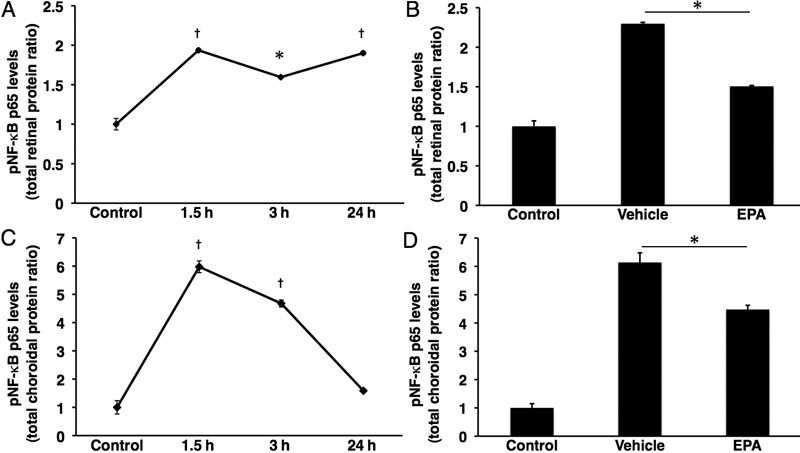
Impact of EPA on pNF-κB in the retina and RPE-choroid complex after LPS injection. **A**: Time course of pNF-κB p65 in the Retina. Values are means±SEM (n=6 at each time point). **B**: Bars represent the protein level of pNF-κB p65 in retinal tissue, 1.5 h after LPS injection. Values are means±SEM (n=4 to 10). **C**: Time course of pNF-κB p65 in the RPE-choroid complex. Values are means±SEM (n=6 at each time point). **D**: Bars represent the protein level of pNF-κB p65 in the RPE-choroid complex, 1.5 h after LPS injection. Values are means±SEM (n=4 to 10). †p<0.01 and *p<0.05.

To further explore the mechanism by which EPA suppressed acute ocular inflammation, we examined NF-κB activation by measuring the pNF-κB p65 protein level in the retina and the RPE-choroid complex (Figure 5). As a preliminary experiment, we first sought the time course of phosphorylation of the NF-κB p65 protein during EIU. After LPS stimulation, the pNF-κB p65 level increased, peaked at 1.5 h, and reached a plateau in the retina (Figure 5A). Likewise, in the RPE-choroid complex, the pNF-κB p65 protein level peaked at 1.5 h; however, unlike in the retina it returned to the base value at 24 h after LPS injection (Figure 5C). The data indicated that the time point to evaluate EPA-treatment on NF-κB activation was 1.5 h after LPS injection.

At 1.5 h after LPS injection, retinal NF-κB activation was significantly increased in vehicle-treated EIU animals compared with control animals (n=4 to 10, p<0.01; Figure 5B). However, EPA administration reduced the phosphorylation of NF-κB p65 protein by 34.5% in the retinal tissues of EIU animals (n=10, p<0.05; Figure 5B). In the RPE-choroid complex, EPA administration decreased the pNF-κB p65 protein by 27.1% compared with EIU animals (n=4 to 10, p<0.05; Figure 5D).

## Discussion

In the current study, we describe the therapeutic effect of EPA on acute ocular inflammation using an animal model of experimental uveitis. EPA suppresses the expression of pro-inflammatory cytokines and reduces the level of pNF-κB p65 in the posterior segment of the eye. To our knowledge, this is the first report of in vivo evidence regarding the protective effect of ω-3 PUFA during acute retinal and choroidal inflammation.

It has been reported that leukocytes are markedly attracted to inflamed ocular tissues such as the iris [23,24], vitreous cavity [22,25], and retina [26,27] in the EIU model. Consistent with the previous studies, we showed that LPS-induced acute inflammation caused the increase of leukocyte recruitment into the eye. In the current study, EPA treatment reduced the accumulation of adherent leukocytes to the retinal vessels by 43.4% and infiltrated leukocytes into the vitreous cavity by 49.2%, suggesting that EPA suppresses the leukocyte recruitment cascade, at least, earlier than the adhesion step. Previously, it was reported that EPA decreased neutrophil infiltration in an animal model of contact dermatitis [28] and neutrophil transmigration through cultured endothelial cells [29]. In addition, EPA significantly attenuated macrophage infiltration into inflamed pancreatic parenchyma in a model of experimental acute edematous pancreatitis [30]. Neutrophils and macrophages are major leukocyte constituents recruited into the ocular tissues during EIU [21,31]. Therefore, previous and present data indicate the potential of EPA as a therapeutic strategy against uveitis via blockade of the leukocyte recruitment cascade.

The anti-inflammatory aspects of ω-3 PUFAs are also related to the regulation of inflammatory cytokines [32]. In the current study, we investigated the impact of EPA supplementation on the production levels of selected members of these inflammation-associated molecules during experimental ocular inflammation. Our data showed that systemic administration of EPA significantly decreased the protein levels of the pro-inflammatory cytokine IL-6 and potent macrophage-recruiting chemokine MCP-1 in the inflamed retinas and RPE-choroid complexes. IL-6 plays a key role in the pathogenesis of EIU, in concert with inflammatory cytokines TNF-α and IL-1β [33-35]. MCP-1 is an important mediator of monocyte infiltration [36], which is upregulated during EIU [35,37,38]. It was previously documented that infiltrated leukocytes were the cellular source of IL-6 [34] and MCP-1 [38] in ocular tissues. Thus, attenuation of leukocyte recruitment by EPA may result in the decreased production levels of IL-6 and MCP-1 in the retina and choroid.

Interestingly, in this study the level of the ICAM-1 protein, an endothelial adhesion molecule regulating leukocyte recruitment, was not decreased by EPA in the inflamed ocular tissues. The protein level of retinal ICAM-1 in EPA-treated EIU animals did show a trend to lower levels compared with vehicle-treated EIU animals, however the difference did not reach statistical significance. During inflammation leukocytes are recruited in a cascade-like fashion, starting with rolling, followed by firm adhesion and extravasation. In addition, ICAM-1 is known to be a key molecule upregulated in the pathogenesis of EIU [22,35]. Thus, previous reports demonstrated the reduction of ICAM-1 expression by therapeutic intervention, and it was concluded in the studies that this reduction resulted in attenuation of leukocyte adhesion and/or transmigration [35,39]. By contrast, the current data suggest that EPA affects the leukocyte recruitment by a different pathway. In fact, Tull et al. showed that EPA had no ability to decrease adhesion molecules such as E-selectin and ICAM-1, representatives of endothelial surface adhesion molecules, in cultured endothelial cells stimulated with TNF-α, yet EPA reduced the neutrophil transmigration in their study [29]. Instead, they demonstrated that EPA reduced CD11b on the surface of neutrophils [29]. CD11b is a component of the leukocyte adhesion molecule Mac-1 (CD11b/CD18), which is a β2 integrin family member that binds to ICAM-1 and is expressed on the surface of neutrophils and macrophages [40,41]. This indicates that EPA may reduce the leukocyte recruitment by targeting leukocyte surface adhesion molecules, but not endothelial surface adhesion molecules. It will be important to explore the effect of EPA on endothelial and leukocyte surface adhesion molecules in vivo, e.g., in the EIU model.

We demonstrated that the level of pNF-κB p65 was significantly suppressed by EPA in the inflamed retina and choroid. LPS downstream signaling leads to the activation of NF-κB [42]. NF-κB regulates various inflammatory processes [43], including transcription of the *IL-6* [44] and *MCP-1* genes [45,46]. Recent studies have shown that ω-3 PUFA downregulates the activity of NF-κB [47]. Therefore, in this study it seems that EPA decreases the secretion of inflammatory factors such as IL-6 and MCP-1 via suppression of NF-κB activation.

In summary, we show that systemic treatment of EPA effectively suppresses acute ocular inflammation in the EIU model. Specifically EPA reduces leukocyte recruitment to the vitreous cavity and retina during acute ocular inflammation. The attenuation of leukocyte recruitment might lead to the reduction of secretion of IL-6 and MCP-1. In addition, EPA also suppressed NF-κB activation, which regulates *IL-6* and *MCP-1* transcription. Thus, it may be that EPA has a reciprocal pathway to decrease the levels of pro-inflammatory cytokines via regulation of NF-κB activation and leukocyte recruitment. Our data suggest EPA, a constituent of fish oil, may be beneficial in the prevention and treatment of uveitis.
